# Spatial transfer of object-based statistical learning

**DOI:** 10.3758/s13414-024-02852-3

**Published:** 2024-02-05

**Authors:** Dirk van Moorselaar, Jan Theeuwes

**Affiliations:** 1 Department of Experimental and Applied Psychology, Vrije Universiteit Amsterdam, Amsterdam, the Netherlands; 2 Institute of Brain and Behaviour Amsterdam (iBBA), Amsterdam, the Netherlands; 3 William James Centre for Research, ISPA-Instituto Universitario, Lisbon, Portugal

**Keywords:** Attention, Object-based, Attention in learning, Visual search

## Abstract

A large number of recent studies have demonstrated that efficient attentional selection depends to a large extent on the ability to extract regularities present in the environment. Through statistical learning, attentional selection is facilitated by directing attention to locations in space that were relevant in the past while suppressing locations that previously were distracting. The current study shows that we are not only able to learn to prioritize locations in space but also locations within objects independent of space. Participants learned that within a specific object, particular locations within the object were more likely to contain relevant information than other locations. The current results show that this learned prioritization was bound to the object as the learned bias to prioritize a specific location within the object stayed in place even when the object moved to a completely different location in space. We conclude that in addition to spatial attention prioritization of locations in space, it is also possible to learn to prioritize relevant locations within specific objects. The current findings have implications for the inferred spatial priority map of attentional weights as this map cannot be strictly retinotopically organized.

## Introduction

It is well established that humans can learn visual patterns that are defined statistically or probabilistically, such as objects that co-occur frequently (Fiser & Aslin, 2001; Saffran rt al., 1996). This type of learning is called ‘‘statistical’’ as it is assumed to take place implicitly, automatically, and without intention to learn (Turk-Browne et al., 2005). A surge in recent studies revealed that visual statistical learning also plays a crucial role in attentional selection (for a review, see Theeuwes et al., 2022). For example, studies have shown that when the target is more likely to appear within particular locations or quadrants within a search display, participants are faster to find the target when it is presented at high-probability locations than when presented at low-probability locations (Chun & Jiang, 1998; Ferrante et al., 2018; Geng & Behrmann, 2005; Huang et al., 2022). The idea is that through past learning experiences, attentional selection is facilitated by directing attention to objects and events that were relevant in the past while suppressing objects and events that previously were distracting (Theeuwes, 2019).

The effect of selection history on attentional selection demonstrates that selection is not, as traditionally believed, solely determined by the interaction between top-down and bottom-up processes (Awh et al., 2012; Luck et al., 2021). To date, however, studies investigating the effects of selection history on attentional selection have mainly focused on learning the distributional properties in time and space of objects within the environment (Theeuwes et al., 2022). This raises the question how, if at all, learning regularities regarding the locations of key parts *within* objects also influences attentional selection.

Previous research has demonstrated the coexistence of space-based attention and object-based attention (Egly et al., 1994). Object-based attention studies have shown that when attention is directed to a part of an object, such as through a spatial cue, other parts of that object benefit perceptually (Moore et al., 1998; Watson & Kramer, 1999). However, it is noteworthy that, unlike the robust effects observed with space-based attention, object-based attention effects are typically smaller and more prone to variation (Reppa et al., 2012). Specifically, object-based effects seem to be contingent on conditions where the task requires attention to be distributed across multiple locations on the screen. This can occur, for instance, when attention is directed to both cued and uncued locations (Egly et al., 1994; Moore et al., 1998) or when the display contains multiple targets (Watson & Kramer, 1999). Conversely, when attention is highly focused on a single location, object-based effects are not observed (Shomstein & Yantis, 2002). While previous research on object-based attention has provided important insights into the limits of object-based effects, it has mainly centred on the traditional attentional selection dichotomy, regarding top-down factors (Drummond & Shomstein, 2010; Kravitz & Behrmann, 2008) and bottom-up factors, such as surface characteristics, geometric discontinuities (Watson & Kramer, 1999), and object orientation (Al-Janabi & Greenberg, 2016). Yet, previous studies investigating the influence of statistical regularities on object-based attention mainly did so by making the regularity contingent on the position of a spatial cue (Chou & Yeh, 2018; Nah & Shomstein, 2020), leaving it unclear whether learning can serve to prioritize parts of specific objects.

One recent study that investigated these issues showed that it was not only possible to learn to prioritize specific locations within a search display, but also to learn to prioritize specific locations *within* objects. In this study by van Moorselaar and Theeuwes (2023), participants were confronted with everyday objects (a hammer or a shoe) presented at the centre of the display. During a learning phase, participants learned to prioritize a particular location within an object (e.g., the head of hammer, or the heel of the shoe) because the target (the letter T) was more likely to be found at these locations than at other locations within the object. Critically, after learning, when these objects were rotated 45° away from the orientation during which learning took place, the learned attentional bias regarding a particular location remained in place even though the target was now equally likely to appear at each location within the object. This study highlights that, akin to spatial attention, object-based attention is not exclusively influenced by top-down and bottom-up factors, but is also remarkably sensitive to regularities across displays such that participants can learn and consistently prioritize specific locations within an object.

**Fig. 1:**
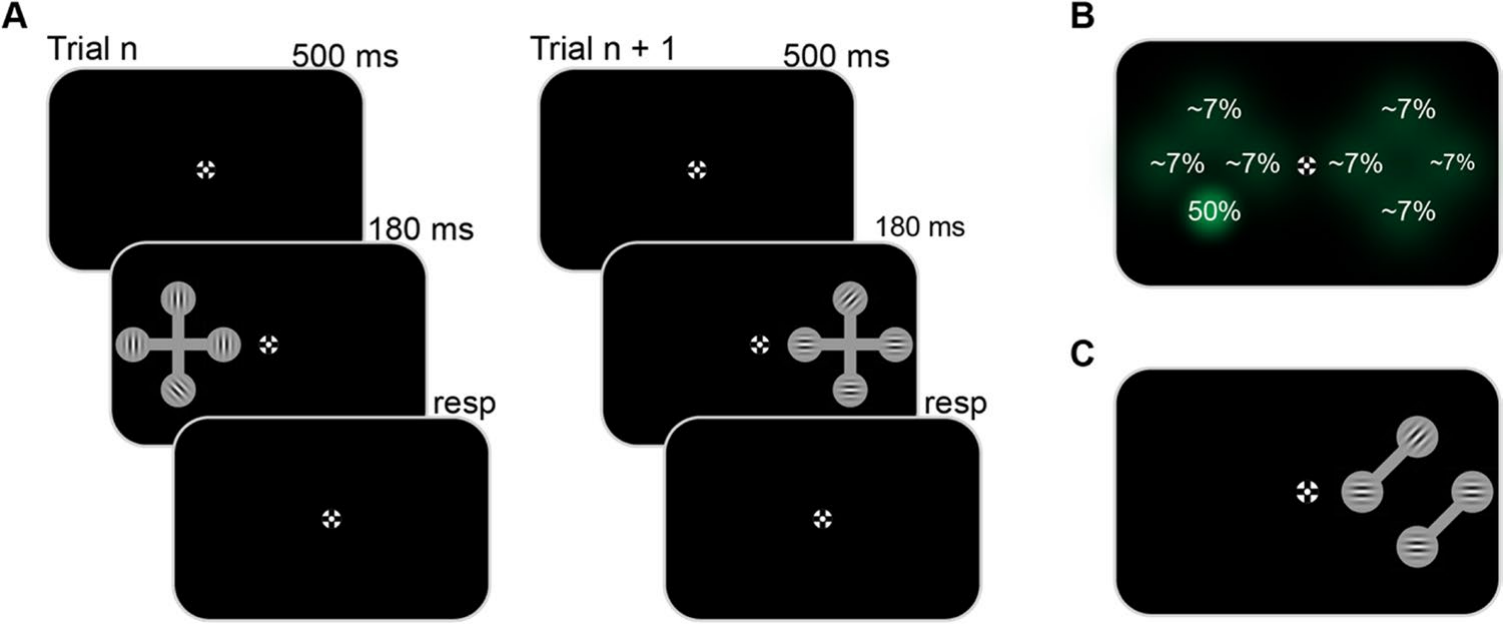
Schematic representation of the experimental procedure in Experiments 1 and 2. On each trial, an artificial object (a barbell-shaped cross) was presented on the left or right of fixation. Embedded within these crosses were four Gabor patches, of which only one had a tilt (left or right), and participants (all *n* = 36) were instructed to respond to the orientation of that tilted Gabor. To induce statistical learning, on one side of the display the target appeared with higher probability at a specific location (i.e., one of the locations on the vertical object axis). (**A**) Example trials in Experiment 1 where the barbell-shaped cross appeared on both sides of fixation. (**B**) Schematic representation of the spatial regularity of the target position across trials, where percentages represent the probability that the tilted Gabor appeared at that location. (**C**) In Experiment 2, at the neutral display side without a spatial imbalance, the Gabors were embedded within two independent objects instead of an integrated object

The current study was designed to determine the boundary conditions of this object-based statistical learning. Instead of using pictures of actual existing objects (which may allow learning to be easier), the current study employed artificial objects. Previous research has shown that such artificial objects lend themselves to inducing object-based attention effects (Al-Janabi & Greenberg, 2016; Kravitz & Behrmann, 2008; Watson & Kramer, 1999), yet there is no evidence that observers are able to learn to prioritize specific parts of such artificial objects. More importantly, the object-based statistical learning effect as reported by Van Moorselaar and Theeuwes (2023) was limited because learning took place for an object presented centrally at fixation, and testing took place by rotating this centrally presented object. Therefore, there is the possibility that the object-based transfer was observed because the object basically stayed on the same location on the retina and was only rotated around its central axes. In the current study, the object appeared at two different locations, while ensuring that learning took place on one side of the fixation point while testing took place at the other side of fixation (see Fig. 1). In other words, we determined whether the learned attention priority within the object stayed in place even when the object appears at a completely different location in space. Such an effect would imply that object-based statistical learning is robust and is tied to the object, and not to the (learned) retinotopic location of the object on the screen.

## Experiment 1: Object-based spatial transfer of learned attentional biases

### Methods

#### Participants

Participants were recruited via the online platform Prolific (www.prolific.co; £3.75). Prior to the experiments, which were conducted online on a JATOS server (Lange et al., 2015), participants provided digital informed consent via Qualtrics (Qualtrics, Provo, UT, USA). Datasets were only analyzed when an experiment was completed in full. The ethics committee of the Faculty of Behavioral and Movement sciences Vrije Universiteit Amsterdam approved the study, which was conducted according to the tenets of the Declaration of Helsinki.

The final sample (N = 36) in Experiment 1 (mean age = 26 years, range = 20–39 years; 18 female) was obtained after replacing one participant who was identified as an outlier (based on overall accuracy; >2.5 *S.D.* from the group mean). Sample size was determined based on a series of pilot experiments (*N* = ~20), where the effect size ranged between 0.4 and 0.5. In combination with an alpha level of 0.05 and a power of .80, G*power (Faul et al., 2007) suggested that a sample size of 32 was sufficient to detect a one-tailed effect.

#### Task, stimuli and procedure

As the experiment was conducted online, we had little control over the experimental setting; for replication purposes we report pixel values to describe the stimuli. The experiment was created in OpenSesame v3 (Mathôt et al., 2012) using OSWEB (version 1.4).

Each trial started with a 500-ms black fixation display, in which a black and white circular fixation point as designed by Thaler et al. (2013) was shown at the center of the screen. Subsequently, a search display appeared, in which a barbell-shaped cross appeared on the left or the right side of the fixation marker (see Fig. 1; centre 235 pixels away from fixation). The four circled placeholders (radius = 44 pixels) within the object were equidistant from the centre of the cross (150 pixels). Embedded within the circular shapes at the edges of this cross were four Gabor patches, of which one, the target, was tilted left or right (45° and 135°; counterbalanced across trials), while the other patches were either all vertically or horizontally oriented (counterbalanced across trials). At one side of the display, the biased side, the target appeared with a higher probability (50%) at either the top or the bottom position in the cross (counterbalanced across participants), whereas on the other side of the display, the neutral side, the target appeared with equal probability across all four possible positions (see Fig. 1B). As a result, the search display appeared on the biased side more frequently (71.4%) than on one the neutral side of the display (counterbalanced across participants). The possible high-probability locations were limited to the vertical axis to match the retinal eccentricity of the high-probability location between left and right barbell crosses. To prevent eye movements during visual search, this display was only visible for 180 ms, an exposure duration that is too short to make directed eye movements (Heeman et al., 2019). During the subsequent response screen, which contained only the fixation marker, participants had to indicate the orientation of the unique Gabor patch. This screen remained visible until response, with a timeout of 2,000 ms. In case of an incorrect response, the fixation point, which remained on-screen for another 250 ms, turned into a red X.

Participants were instructed to keep their eyes at fixation, and to indicate the orientation of the Gabor as fast as possible, while trying to keep the number of errors to a minimum. The experiment consisted of six experimental blocks of 98 trials each, preceded by a series of 20 practice trials, which was repeated until during practice average reaction time (RT) was below 1,500 ms and average accuracy was above 66%. Halfway through each block participants were given the opportunity to take a short break, and at the end of each block they received feedback on their performance (i.e., mean RT and accuracy). After the last block, in a series of questions, participants were asked to indicate whether they noticed that a location contained the target with higher probability (yes or no), which location contained the target with a higher probability (top, left, right, or bottom), whether they noticed that this was only the case on one side of the display (yes, or no), and which side of the display contained the spatial imbalance (left or right).

#### Statistics

Search times analyses were limited to data of correct trials only. RTs were filtered in a two-step trimming procedure: trials with RTs shorter than 200 ms were excluded, after which data were trimmed based on a cutoff value of 2.5 SD from the mean per participant. Exclusion of incorrect responses (11.7%) and data trimming (2.4%) resulted in an overall loss of 14.1% of trials. Before analyzing the results, any trial in which the location of the target repeated from one trial to the next were excluded (25.0%), such that any observed effects could not be explained by intertrial priming (Maljkovic & Nakayama, 1994). Remaining RTs were analyzed with repeated-measures ANOVAs, where reported *p*-values are Greenhouse-Geiser-corrected in case of sphericity violations, followed by planned comparisons with paired t-tests using JASP software (Wagenmakers, 2018).

### Results and discussion

**Fig. 2:**
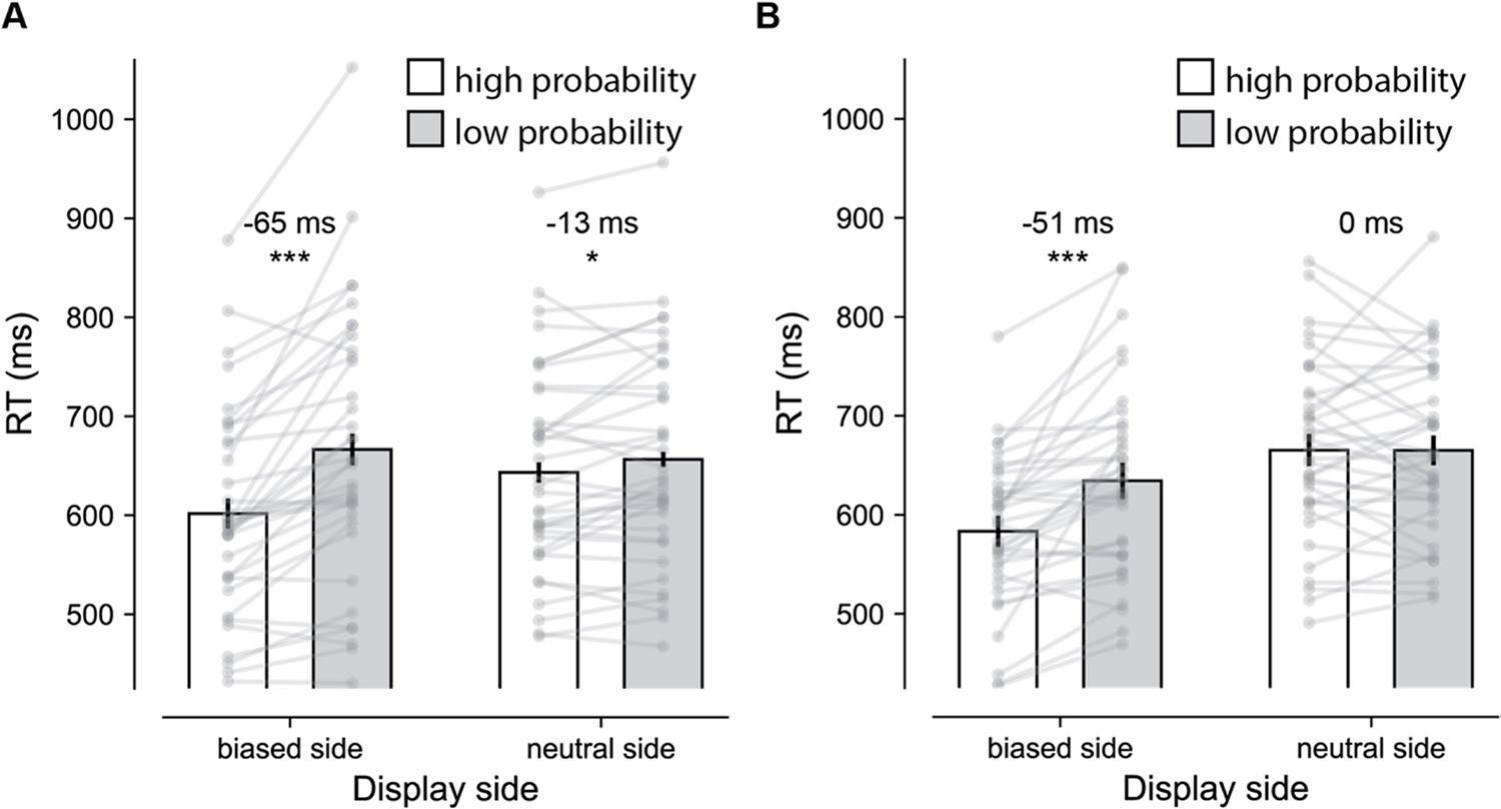
Learned attentional prioritization is not bound to a specific location in space but can be attached to a particular location within an object. (**A**) Experiment 1: Object-based transfer of statistical learning. Participants detected targets faster at high (white bar) versus low (grey bar) probability object locations, not only at the biased side that contained the regularity (*m*_high_ = 601.7; *m*_low_ = 666.5; Δ*m = 65.; n = 36;* two-tailed *p* < 0.001; *d* = 1.0; 95% CI = -86.7 – -42.7), but also, albeit attenuated, at the neutral side without a regularity (*m*_high_ = 643.2; *m*_low_ = 656.4; Δ*m = 13.; n = 36;* two-tailed *p* = 0.023; *d* = 0.40; 95% CI = -24.4 – -1.88). (**B**) Experiment 2: No spatial transfer of the learned effect (*m*_high_ = 583.4; *m*_low_ = 634.4; Δ*m = 51.; n = 36;* two-tailed *p* < 0.001; *d* = 0.84; 95% CI = -71.5 – -30.5) when the target was embedded in one of two independent objects at the neutral side (*m*_high_ = 665.4; *m*_low_ = 665.1; Δ*m = 0.; n = 36;* two-tailed *p* = 0.98; *d* = 0.004; 95% CI = -17.5 – 18.0). The height of each bar reflects the population average, and error bars represent 95% within-subject confidence intervals (Morey, 2008). Data from each participant are represented as grey dots

To examine whether the high-probability location was enhanced, and, critically, whether this learned prioritization stayed in place when the object appeared at the other side of fixation, we conducted a repeated-measures ANOVA with within subject’s factor Display side (biased, neutral) and Target position (high probability, low probability), where on the neutral side locations were artificially coded as high and low probability based on the spatial imbalance on the biased side. As visualized in Fig. 2A, observers were reliably faster to detect a target at the high-probability location at both the biased side containing the actual regularity and at the neutral side without a spatial imbalance (main effect Target position: *F* (1, 35) = 29.61, *p* < 0.001, ${n}_{p}^{2}$ = 0.46). Nevertheless, a reliable interaction indicated that while the learned effect appeared to generalize to the neutral display side, it was reliably less pronounced without an actual spatial imbalance (*F* (1, 35) = 29.26, *p* < 0.001, ${n}_{p}^{2}$ = 0.46). Indeed, planned pairwise comparison confirmed that the effect was evident at both display sides, but more pronounced at the biased side (*t* (35) = 5.98, *p* < 0.001, *d* = 1.00) than at the neutral side (*t* (35) = 2.37, *p* = 0.023, *d* = 0.40). Analysis on mean accuracy (*M =* 87%, *SD* = 9.1) mimicked the main findings, although in addition to the main effect of Target position (*F* (1, 35) = 16.31, *p* < 0.001, ${n}_{p}^{2}$ = 0.32), there was no significant interaction (*F* = 0.56, *p* = 0.46).

Although the observed modulation at the neutral side is consistent with object-based statistical learning, it should be noted that at the biased side the effect was substantially more pronounced. Arguably, this is the case because at the biased side the benefit at the high-probability location is a mixture of two effects, object-based learning on the one hand, and high-probability location learning on the other hand. By contrast, the effect at the neutral side reflects the pure object-based effects as both locations within the object at that side of the display contained the target with equal probability. However, we should be careful comparing different studies with different designs; in this respect it is noteworthy that the van Moorselaar and Theeuwes (2023) study observed object-based effects of similar magnitude (~10 ms).

After having established that the learned prioritization appeared to generalize, albeit attenuated, to another spatial location without an increased target probability, we explored to what extent this effect was modulated by participants’ explicit knowledge. Out of the 25 participants who indicated that they noticed the regularity, 13 participants correctly identified both the correct location and display side. However, as a between-subject’s factor, Awareness (13 aware, 23 unaware) did not interact with either the effect of Target position or the Target position by Display side interaction (all *F*s < 1.22, all *p*s > 0.28), suggesting that the effect was not modulated by explicit knowledge of the underlying regularity.

The pattern of results is consistent with the idea that observers learned to prioritize a location within an object that has a higher probability of containing relevant information, and this learned bias stays in place when that object appears at another location in space (i.e., the other side relative to fixation). Nevertheless, this conclusion is premature as the results could also be explained by assuming that participants did not learn to prioritize a specific part of the object, but instead prioritized the bottom or the top side of the display independent of object position. This would imply that the effect observed has nothing to do with object-based attention, but instead represents a learned bias to direct attention to a specific subset of space. Experiment 2 was designed to determine whether the observed effect reflects genuine object-based learning or instead can be explained by a learned attentional bias to either the top or the bottom of the display. For this purpose, at the neutral side the Gabor patches were still presented at the same retinal positions, but the barbells were split such that they no longer formed an integrated object (see Fig. 1C).

## Experiment 2: Object-based spatial transfer cannot be explained by a general spatial bias

Experiment 2 was identical to Experiment 1 except that the object in which learning took place did not stay intact when it appeared at the neutral display side (see Fig. 1C). If participants have learned to either bias the top or the bottom of the display independent of the object presented, there should again be a benefit at the matching high-probability location on the neutral side. If, however, the effect seen in Experiment 1 is truly dependent on prioritization within the object, then we should see no transfer of the bias as that object is no longer present.

### Methods

The methods of Experiment 2 were identical to Experiment 1, except for the following changes. The final sample (N = 36; mean age = 25 years, range = 19–35; 13 female; data of two subjects missing) was obtained after replacing one participant who was identified as an outlier based on accuracy. While the same barbell-shaped cross was presented at the biased side, at the neutral side both barbells were rotated 45° such that they formed two independent objects. Exclusion of incorrect responses (14.3%) and data trimming (2.4%) resulted in an overall loss of 16.6% of trials. Subsequent removal of trials in which the exact target location repeated resulted in another loss of 25.2% of data.

### Results

As visualized in Fig. 2B, participants again learned to prioritize the high-probability location on the biased side, again resulting in a main effect of Display side (*F* (1, 35) = 56.55, *p* < 0.001, ${n}_{p}^{2}$ = 0.62). Critically, this effect no longer appeared to generalize to the neutral side (interaction: *F* (1, 35) = 32.613, *p* < 0.001, ${n}_{p}^{2}$ = 0.48). Indeed, while the difference between high- and low-probability locations was evident at the biased side (*t* (35) = 5.05, *p* < 0.001, *d* = 0.84), it was no longer reliable at the neutral side (*t* = 0.026, *p* = 0.98; BF_01_ = 5.58). As in Experiment 1, the same analysis on mean accuracy (*M =* 82%, *SD* = 9.8) yielded the same pattern of results with a reliable Display side by Target position interaction reflecting that the observed benefit at the biased side did not generalize to the neutral side (*F* (1, 35) = 6.69, *p* = 0.014, ${n}_{p}^{2}$ = 0.16), demonstrating that the observed pattern of results cannot be attributed to a speed-accuracy trade off.

Out of the 24 participants who indicated that they noticed the regularity, 17 participants correctly identified both the correct location and display side. However, as in Experiment 1, including awareness did not modulate any of the observed effects (all *F*s < 2.26, all *p*s > 0.14), suggesting that the effect was again not modulated by explicit knowledge of the underlying regularity.

#### General discussion

Recent research has demonstrated that the implicit extraction of environmental regularities, known as statistical learning, not only changes attentional priorities at given locations in space (Chun & Jiang, 1998; Geng & Behrmann, 2005; Van Moorselaar & Theeuwes, 2021; Wang & Theeuwes, 2018), but can also develop preferential biases for specific parts of an object. In the first demonstration of such object-specific statistical learning, it was shown that attentional prioritization remained bound to a location within an object when it rotated along its central axis (van Moorselaar & Theeuwes, 2023). Here, we show that the learned effect remained bound to the object even when the object appeared at a completely new location within the display. The results show that the learned bias survived the shift to a new display configuration, but only when the object stayed intact at the neutral display location. Together these findings demonstrate that in addition to space-based statistical learning, the visual system can also generate attentional biases independent of the spatial coordinates of the specific object in space.

While it has been suggested that space-based and object-based attention can coexist, the latter seems to manifest primarily in the absence of an alternative strategy. Numerous studies have consistently indicated that object-based effects diminish when attention is no longer diffusely spread across the display but is instead focused with certainty on a specific location or part of an object (Goldsmith & Yeari, 2003; Lavie & Driver, 1996). For instance, Drummond and Shomstein (2010) observed no object-based effects anymore when the upcoming target position became entirely predictable (see also Shomstein & Yantis, 2002). In our current study, attention, by design, was diffuse, with targets appearing unpredictably on both sides of fixation and at different parts of the object. The results of the present investigation underscore that under such conditions, object-based attention can indeed be modulated by statistical learning. This modulation results in the prioritization of specific parts within an object, irrespective of the spatial location where the object appears.

Although the current results confirm that in addition to space-based learning, object-based statistical learning can also tune attentional priorities in response to regularities within the environment, the underlying mechanisms are likely to be different. When applying space-based learning, the attentional effects affect the weights within the assumed spatial priority map such that a location that is likely to contain a target is proactively up-regulated and a location that is likely to contain a distractor is proactively down-regulated (Huang et al., 2022). Critically, it was shown that these weight changes were implemented well before display onset (Huang et al., 2023; Wang et al., 2019), possibly affecting latent neural mechanisms instantiated in short-term synaptic plasticity (van Moorselaar & Slagter, 2020). Consistent with this view, a recent study using MEG showed that neural excitability in early visual cortex regions associated with higher distractor probabilities was reduced during the interval preceding search display onset (Ferrante et al., 2023) (see Zhang et al., 2022, for similar findings using fMRI). Similarly, a study by Duncan et al. (2023) revealed that inserting neutral visual pings in between search display could reveal the otherwise hidden priority landscape, reflecting spatially tuned enhancement of high-probability target locations. All these findings point to proactive changes of the weights of the assumed priority map of space (Theeuwes et al., 2022).

When considering the difference between spatial and object-based learning, it is clear that space-based statistical learning can operate in retinotopic (eye-centered) coordinates. Because the learned attentional priority remains at the same retinotopic location, it is possible to set the attentional weights within the priority map proactively (either by active tuning or within a latent network). Consistent with such a proactive account, a recent study using MEG showed that neural excitability in early visual cortex regions associated with higher distractor probabilities was reduced during the interval preceding search display onset (Ferrante et al., 2023) (see Zhang et al., 2022, for similar findings using fMRI). Similarly, a study by Duncan et al. (2023) revealed that inserting neutral visual pings in between the search display could reveal the otherwise hidden priority landscape reflecting spatially tuned enhancement of high-probability target locations. Attentional prioritizing of a subpart of an object (e.g., the top of the bar bell) can also be done within retinotopic coordinates as long as the object remains static. However, as soon as the object moves to another location on the retina, the prioritization of the subpart of the object can no longer be done in retinotopic coordinates, and needs to be tied to the object itself. Clearly, this prioritization can only be done reactively, that is after the presentation of the learned object. Unlike (retinotopic) space-based learning where the learned bias can be encoded within early visual areas, we have to assume that information from higher cortical areas such as the ventral intraparietal area (e.g., Duhamel et al., 1997), which are known to represent information in object-based coordinates, is fed back to early visual areas to generate attentional prioritization of specific subparts of the learned objects (see also van Moorselaar & Theeuwes, 2023).

To date, paradigms that investigated space-based statistical learning typically presented the target and/or distractor at the very same retinotopic (eye-centered) location. This implies that changes within the spatial priority map could in principle have been exclusively implemented within a retinotopic (eye-centered) coordination system. For example, it is generally assumed that perceptual learning that is an experience-induced gain in discriminating sensory information is assumed to be to be highly specific to the trained retinal location (Karni & Sagi, 1991). Consistent with this, a study by Jiang et al. (2014) showed that after a 90° change in viewpoint, the incidentally learned attentional bias remained tuned to the retinotopic (eye-centered) location and hence did not adjust to the spatiotopic coordinates. By contrast, object-specific tuning as observed here is encoded relative to the external world, as the bias to a location within the object stayed in place when the object was presented at the other side of fixation. This implies that attentional priority is adjusted to the statistical regularities that exist within an object, irrespective of the position of that object within space (as we show here), or the specific orientation of that object (van Moorselaar & Theeuwes, 2023). It thus appears that statistical learning can adjust attentional priorities via distinct neural mechanisms. Whereas space-based statistical learning is retinotopic and proactive, organized by changing synaptic weights in early visual areas (Chelazzi et al., 2014; Duncan et al., 2023; Sprague & Serences, 2013), object-based statistical learning is encoded relative to the external world in higher-order visual areas, and therefore has to be reactive.

The observation that statistical learning can tune attentional priorities via both retinotopic and object-centered representations is consistent with previous research demonstrating that retinotopic and object-centered representations can coexist (Theeuwes et al., 2013; Tipper et al., 1999). This attentional tuning via distinct learning systems also explains why in the current design learning was more pronounced at the biased side. While at the biased side learning could occur within both retinotopic and object-centered representations, the effect at the neutral-side was exclusively driven by object-based statistical learning.

While the current study shows that under rigorous controlled conditions statistical learning within objects can be demonstrated, it is not surprising that we have such an ability, as it is extremely helpful when interacting with real-world objects. Indeed, if we have learned that the switch to turn on our laptop is located at the top left of the machine, we easily will find this location if the laptop moves, rotates, and turns to other locations in space.
